# Analysis of the current situation and influencing factors of nurse change fatigue

**DOI:** 10.3389/fpubh.2025.1566534

**Published:** 2025-04-09

**Authors:** Ming Yu, Jing Ji, Dongying Kan, Dandan Gu, Xixi Yin, Suping Cai, Ronghui Geng, Lingling Jiang

**Affiliations:** ^1^Department of Nursing, Affiliated Rudong Hospital of Xinglin College, Nantong University, Nantong, Jiangsu, China; ^2^Nursing Department, Nantong Tongzhou Hospital of Traditional Chinese Medicine, Nantong, Jiangsu, China

**Keywords:** clinical nurse, change fatigue, emotional intelligence, care perception, influencing factors

## Abstract

**Background:**

As the healthcare environment undergoes rapid evolution, nurses frequently confront multifaceted changes, resulting in significant change fatigue. This fatigue adversely impacts their well-being, diminishes job satisfaction, elevates turnover rates, and undermines the quality of care provided.

**Aim:**

To comprehend the fatigue levels experienced by clinical nurses amidst environmental changes and to analyze the factors influencing this fatigue.

**Methods:**

A survey was conducted among 451 clinical nurses at a tertiary general hospital in Nantong City, utilizing the *Change Fatigue Scale, Care Perception Scale,* and *Emotional Intelligence Scale*.

**Results:**

The change fatigue score was 19.01 ± 3.63, indicating a moderate level of fatigue. Change fatigue was inversely correlated with emotional intelligence and care perception (all *p* < 0.05). Multiple linear stepwise regression analysis revealed that monthly income, professional title, years of work, number of night shifts, care perception, and emotional intelligence were significant factors influencing the change fatigue experienced by clinical nurses amidst environmental changes (all *p* < 0.05). These factors collectively accounted for 76.1% of the total variation.

**Conclusion:**

Clinical nurses exhibit moderate levels of change fatigue. Therefore, it is recommended that nursing managers implement regular emotional intelligence training and reduce night shifts to actively address the rapid changes brought about by the healthcare industry and, consequently, enhance the quality of nursing care.

## Background

In the rapidly evolving medical environment, hospital relocation represents not merely the transformation of physical space but also a profound process involving workflow restructuring and the psychological adjustment of personnel (1). As a crucial component of the medical service system, the physical and mental health status of clinical nurses is intimately tied to the quality of nursing care and patient safety (2). However, the substantial changes brought about by hospital relocation often propel nurses into a psychological dilemma termed “change fatigue,” which stems not only from the rapid alterations in the working environment but also encompasses adjustments in workflow, teamwork, patient management, and other facets (3).While emotional intelligence is generally considered beneficial, recent studies suggest that the relationship between emotional intelligence and fatigue may not be linear. For instance, Partido and Owen (4) found that while emotional intelligence is linked to stress and burnout, excessive self-regulation of emotions can become a fatigue-inducing factor. Similarly, Griffin and Howard (5) proposed that individual differences in emotion regulation may lead to distinct stress patterns, such as cardiovascular reactivity, indicating that over-regulation of emotions might exacerbate fatigue rather than alleviate it.

Change fatigue is a prevalent psychological response among employees undergoing organizational change. It manifests as persistent stress, exhaustion, and fatigue. In severe cases, it may result in diminished work efficiency, increased nursing errors, and even heightened job burnout and turnover rates (6). For clinical nurses, the impact of hospital relocation is particularly pronounced, affecting not only individuals but potentially the entire nursing team.

Emotional intelligence pertains to an individual’s ability to identify, understand, express, and regulate their own and others’ emotions. It is a significant factor influencing an individual’s psychological adjustment capabilities when confronted with pressure and challenges (7). Studies have demonstrated that individuals with high emotional intelligence are better equipped to manage their emotions and maintain a positive attitude, thereby mitigating the risk of change fatigue (8). Therefore, examining the relationship between emotional intelligence and change fatigue among clinical nurses is crucial to understanding how they respond to the changes induced by hospital relocation.

Furthermore, the perception of head nurse care is another pivotal factor influencing nurses’ change fatigue. As the leader of the nursing team, the care and support provided by the head nurse play a vital role in the psychological adjustment and career development of nurses (9). When nurses perceive care and support from their head nurse, they are more inclined to maintain a positive outlook in the face of change, thereby alleviating the severity of change fatigue (10).

Consequently, this study investigated the current situation regarding clinical nurses’ change fatigue, analyzed the influencing factors, and explored their relationship with emotional intelligence and perceptions of head nurse care. The aim was to develop targeted management programs for nursing managers, enabling them to actively respond to the rapid changes in the healthcare industry and, consequently, enhance the quality of care.

## Methods

### Setting

The survey was conducted at the People’s Hospital of Rudong County, located in Nantong City, Jiangsu Province, China. Over the past 3 years, this hospital has undergone a significant transformation and successfully attained the status of a Grade B tertiary general hospital. This remarkable change has not only enhanced the hospital’s hardware facilities and service levels but has also profoundly impacted the nursing staff, particularly by inducing a phenomenon known as “change fatigue.”

### Study design, sample and procedure

From July 2024 to August 2024, clinical nurses working in a tertiary general hospital in Nantong City were selected as the research subjects using the convenience sampling method. Inclusion criteria encompassed clinical registered nurses who had been working in clinical nursing for at least 6 months, had experienced the hospital relocation process, and provided informed consent. Exclusion criteria included training and practice nurses, as well as non-clinical workers such as administrative staff. This study was approved by the Ethics Committee of Rudong County People’s Hospital of Nantong City (Approval No. 2022001).

After obtaining approval from the hospital authority, the investigator, with the assistance of the hospital’s nursing director, explained the purpose of the study to the nurses who met the inclusion criteria and obtained their informed consent. The nurses then completed the questionnaire anonymously. On-site, the investigator verified the completeness of the questionnaire content, promptly supplemented any omissions, and confirmed and corrected any logical errors.

### Questionnaire

#### General information questionnaire

Gather and document the personal information of clinical nurses, which includes their age, gender, educational background, professional title, years of clinical work experience, monthly frequency of night shifts, level of job satisfaction, willingness to resign, and participation in humanistic care training, among other relevant details.

#### Care factors survey-care sense questionnaire (caring factor survey-caring of manager, CFS-CM)

The CFS-CM was utilized to evaluate the level of care perceived by the head nurse over the past 2 weeks to 1 month (9). The scale comprises 10 items, employing the Likert 7-point scoring method, where 1 represents “very disagree” and 7 signifies “very agree.” A higher total score indicates a greater appreciation by the nurse for the role of the head nurse in nursing care. The Cronbach’s alpha coefficient for this scale was 0.961, and in the present study, it was found to be 0.983.

#### Emotional intelligence scale (Wong law emotional intelligence scale, WLEIS)

Yefei Wang developed the Chinese Workplace Emotional Intelligence Scale (WLEIS), which assesses the emotional intelligence of research subjects within a specific work environment (11). This scale consists of 16 items divided into four dimensions: self-emotion perception, others’ emotion perception, emotion management, and emotional application, with four items in each dimension. Utilizing the Likert 7-point scoring method, the score range is from 0 to 96 points. A higher score indicates a higher level of individual emotional intelligence. The overall Cronbach’s *α* coefficient for the scale was 0.860. In this study, the Cronbach’s α coefficient for the emotional intelligence scale was 0.916. Specifically, the Cronbach’s *α* coefficients for the self-emotion perception, others’ emotion perception, emotion management (referred to here as emotional control for clarity), and emotional application dimensions were 0.740, 0.778, 0.736, and 0.724, respectively.

#### Six change fatigue measurement scale (the six-item change fatigue measurement scale)

As developed by Beernerth in 2011, the Six Change Fatigue Measurement Scales are designed to assess change fatigue among clinical nurses who have undergone changes in the hospital environment (2). This scale consists of 6 items, with scores ranging from 1 to 7 points, corresponding to “strongly disagree” to “strongly agree,” resulting in a total score range of 6–42 points. A higher score indicates more severe change fatigue. The Cronbach’s *α* coefficient for the scale was 0.85, and in this study, it was found to be 0.910.

### Statistical method

When items within a scale were missing, a total score was calculated only if at least half of the questions were answered. For the missing items, the average score of the other items within the scale was assigned. The percentage of estimated items relative to the total sample was X%, which falls within the acceptable margin for estimation. The reliability of the scales was assessed using the Cronbach’s *α* internal consistency coefficient. The distribution of sample characteristics and discontent regarding change fatigue was evaluated for all study respondents. Descriptive statistics were presented in terms of frequencies, mean values, and standard deviations (SDs).

The correlation analysis was performed using Pearson’s correlation coefficient to assess the relationships between change fatigue, care perception, and emotional intelligence. A *p*-value of less than 0.05 was considered statistically significant. Logistic regression analysis was employed to identify the characteristics of those who exhibited high discontent with their change fatigue. Results were deemed statistically significant at a *p*-value of less than 0.05, using two-tailed tests. Statistical analyses were conducted using SPSS version 26.0.

## Results

### Respondent characteristics

In this study, a total of 451 questionnaires were distributed, and all 451 were collected as valid responses, resulting in an effective recovery rate of 100%. The demographic information is presented in Table 1.

**Table 1:** Demographic characteristics of clinical nurses (*n* = 451).

Variable	*N*	Percentage (%)
Age		
≤25 years	70	15.50%
26–30 years	126	27.90%
31–40 years	193	42.80%
>40 years	62	13.80%
Gender		
Male	2	0.40%
Female	449	99.60%
Educational background		
Junior college	139	30.80%
Bachelor’s degree or above	312	69.20%
Professional title		
Nurse and below	253	56.10%
Nurse in charge and above	198	43.90%
Years of experience		
1–3 years	138	30.60%
4–6 years	82	18.20%
7–10 years	70	15.50%
11–15 years	65	14.40%
>15 years	96	21.30%
Monthly income (RMB)		
<4,000	79	17.50%
4,001–6,000	150	33.30%
6,001–8,000	72	16.00%
>8,000	150	33.30%

### Descriptive results

The change fatigue score of clinical nurses was 19.01 ± 3.63, indicating a moderate level of fatigue. Change fatigue was inversely correlated with emotional intelligence and care perception (all *p* < 0.05) (Table 2).

**Table 2:** Change fatigue, care perception, and emotional intelligence scores of clinical nurses (*n* = 451).

Variable	Total score	The entries are equally divided
Self-emotional perception	19.62 ± 3.07	4.90 ± 0.77
Emotional control	17.69 ± 3.76	4.42 ± 0.94
Emotional use	18.43 ± 3.357	4.60 ± 0.84
Other peoples emotional perception	18.65 ± 3.208	4.66 ± 0.80
Total emotional intelligence score	74.39 ± 11.84	4.65 ± 0.74
Care perception	43.08 ± 7.50	6.15 ± 1.07
Change fatigue	19.01 ± 3.63	3.17 ± 0.61

Multiple linear stepwise regression analysis revealed that monthly income, professional title, years of work, number of night shifts, care perception, and emotional intelligence were significant factors influencing the change fatigue experienced by clinical nurses amidst environmental changes (all *p* < 0.05). These factors collectively accounted for 77.6% of the total variation (Table 3).

**Table 3:** Comparison of different characteristics (*n* = 451).

Variable	Change fatigue score (mean ± SD)	Statistics	*p*-value
Gender		*t* = −0.128	0.918
Male	18.91 ± 1.11		
Female	19.02 ± 3.64		
Professional title		*t* = 11.218	<0.05
Nurse and below	20.52 ± 2.71		
Nurse in charge and above	17.09 ± 3.77		
Overtime (past 12 months)		*t* = 4.145	<0.05
Yes	19.17 ± 3.57		
No	15.86 ± 3.57		
Intention to resign		*t* = 4.254	<0.05
Yes	20.11 ± 3.16		
No	18.55 ± 3.73		
Age		*F* = 21.38	<0.05
≤25	20.82 ± 3.11		
26 ~ 30	20.26 ± 2.32		
31 ~ 40	18.02 ± 3.92		
>40	17.56 ± 3.91		
Marital status		*F* = 14.747	<0.05
Unmarried	20.53 ± 3.01		
Married	18.47 ± 3.7		
Divorce/widowed	19.93 ± 2.84		
Educational background		*t* = 5.106	<0.05
Junior college	20.29 ± 3.1		
Bachelor’s degree or above	18.45 ± 3.71		
Years of experience		*F* = 31.615	<0.05
1–3 years	20.15 ± 3.1		
4–6 years	20.07 ± 2.3		
7–10 years	20.74 ± 3.06		
11–15 years	17.15 ± 3.43		
>15 years	16.49 ± 3.95		
Number of children		*F* = 14.55	<0.05
No children	18.12 ± 3.99		
One child	19.96 ± 3.01		
Two or more children	19.48 ± 2.85		
Monthly income (RMB)		*F* = 72.911	<0.05
<4,000	21.45 ± 2.62		
4,001 ~ 6,000	20.21 ± 2.75		
6,001 ~ 8,000	19.82 ± 2.67		
>8,000	16.16 ± 3.49		
Monthly night shifts		*F* = 35.257	<0.05
0	16.04 ± 3.63		
1 ~ 3	19.78 ± 2.88		
4 ~ 9	19.86 ± 3.25		
≥10	20.02 ± 2.55		
Preparation for change		*F* = −9.255	<0.05
Yes	17.68 ± 3.8		
No	20.6 ± 2.66		

### Correlation analysis

The results of the correlation analysis revealed significant negative correlations between change fatigue and emotional intelligence (*r* = −0.666, *p* < 0.001) as well as care perception (*r* = −0.765, *p* < 0.001), indicating that higher levels of emotional intelligence and care perception are associated with lower levels of change fatigue (Table 4).

**Table 4:** Correlation coefficient of change fatigue and care perception and emotional intelligence among clinical nurses.

Variable	1	2	3	4	5	6	7
1. Change fatigue	1	−0.644**	−0.595**	−0.562**	−0.556**	−0.666**	−0.765**
2. Self-emotional perception	–	1	0.648**	0.671**	0.642**	0.830**	0.354**
3. Emotional control	–	–	1	0.779**	0.725**	0.903**	0.401**
4. Emotional use	–	–	–	1	0.757**	0.911**	0.372**
5. Other peoples emotional perception	–	–	–	–	1	0.883**	0.326**
6. Emotional intelligence	–	–	–	–	–	1	0.413**
7. Care perception	–	–	–	–	–	–	1

**p < 0.001.

### Multivariate analysis of change fatigue among clinical nurses

Multiple linear regression analysis was conducted with the clinical nurses’ change fatigue score serving as the dependent variable, and variables that exhibited statistically significant differences in the univariate analysis were included as independent variables. The results of the collinearity diagnosis revealed that the variance inflation factor ranged from 1.310 to 2.413, indicating the absence of multicollinearity among the variables. The outcomes of the multiple linear regression analysis showed that monthly income, professional title, years of work experience, number of night shifts, care perception, and emotional intelligence were the primary factors influencing change fatigue among clinical nurses (all *p* < 0.05). These factors collectively accounted for 77.6% of the total variation (Table 5).

**Table 5:** Multiple linear regression analysis of clinical nurses (*N* = 451).

	*β*	Standard error	*β’*	*t*	*p*
(Constant)	39.804	0.821		48.475	0
Care perception	−0.243	0.014	−0.502	−17.648	0
Emotional intelligence	−0.123	0.008	−0.4	−15.457	0
Monthly income	−0.428	0.106	−0.131	−4.047	0
Professional ranks and titles	−0.98	0.261	−0.134	−3.751	0
Working life	0.268	0.083	0.113	3.243	0.001
Monthly night shift	0.255	0.113	0.062	2.254	0.025

*R2 = 0.764, adjusted for R2 = 0.761; F = 240.155, P < 0.001.*

## Discussion

### Change fatigue of clinical nurses is at a medium level

The change fatigue score of clinical nurses in this study, who experienced environmental changes, was 19.01 ± 3.63, which was higher than the moderate level found in a study by Fernemark et al. (12) among Swedish doctors. This difference may be attributed to the distinct populations of the study subjects. Research indicates that nurses tend to experience change fatigue three times more frequently in the healthcare system (13). In the healthcare system, nurses, as the primary group in direct contact with patients, not only bear the burden of heavy nursing tasks but also need to constantly adapt to new workflows, technological updates, and information system changes.

To alleviate change fatigue among nurses, medical institutions should rationally arrange change plans, provide training and support, strengthen communication, and prioritize the physical and mental health of nurses (6). A review by Brown on the relationship between resilience training and nurse change fatigue suggests that resilience can be a key measure to mitigate change fatigue (14). Additionally, the change diary (a tool for tracking organizational changes), serving as a preventive and predictive tool, can effectively reduce the occurrence of nurse change fatigue by using a weekly timeline to count, evaluate, and coordinate various changes within the organization. This aims to proactively assess and arrange the organization’s change process (14).

### The influencing factors of change fatigue among clinical nurses

#### Monthly income

This study found that the monthly income of clinical nurses was negatively correlated with their change fatigue (*β* = −0.428, *p* < 0.05), indicating that nurses with higher monthly incomes experienced relatively less change fatigue. This result suggests that economic factors play a significant role in nurses’ responses to job changes, and that a higher income may assist nurses in better coping with the stress associated with change and reducing fatigue. Income serves not only as a primary motivator for nurses in their work but also as a reflection of their work value and family economy. The income level directly influences the quality of life of nurses (15). It is recommended that managers optimize the nurse compensation system and increase income to mitigate change fatigue. Additionally, strengthening psychological support and communication is crucial to encourage nurses’ active participation in change decision-making.

#### Professional title

The results of this study demonstrated that the professional title of clinical nurses affects their change fatigue (*β =* −0.98, *p* < 0.05). Specifically, nurses with higher professional titles experienced less change fatigue, which is attributed to their advantages of higher income, attention to career development, rich experience, and strong adaptability. Nurses holding high professional titles typically earn higher incomes, prioritize their career development, possess extensive work experience, and demonstrate robust adaptability, enabling them to more effectively address the challenges posed by job changes. Furthermore, they may possess a more mature psychological state and more efficient coping mechanisms, allowing them to better maintain psychological balance and reduce the stress, fatigue, and burnout associated with change. It is recommended that managers focus on the professional title promotion system, provide nurses with more career development opportunities, and strengthen psychological support to assist nurses at all levels in effectively responding to reforms and maintaining psychological balance.

#### Years of service

The results of this study indicate that the working years of clinical nurses impact their change fatigue (*β =* −0.268, *p* < 0.05). Contrary to what might be expected, a longer working life is associated with heavier change fatigue. This can be attributed to several factors: the accumulation of physical and mental fatigue due to prolonged adaptation to ever-changing work environments, technologies, and processes (16); the increased family responsibilities and personal pressures that accompany the accumulation of work experience, which hinder their ability to adapt to change; long-term intensive work leading to job burnout, thereby diminishing their acceptance and responsiveness to change; and finally, deficiencies in the management and support system during times of change, such as the absence of clear change goals, strategies, and effective communication mechanisms, which further exacerbate nurses’ change fatigue (17). Consequently, managers must implement corresponding measures, such as providing necessary support and resources, optimizing change plans, and enhancing communication mechanisms, to alleviate nurses’ change fatigue.

#### Night shift in months

The results of this study revealed that the number of clinical nurses affects their change fatigue (*β* = 0.255, *p* < 0.05). Similarly, a study by Sarıgül and Uğurluoğlu (13) demonstrated comparable findings, showing that an increase in monthly night shifts leads to heightened fatigue. This may be attributed to night shift work disrupting the normal biological clock, thereby increasing work stress and physiological burden, and making nurses more susceptible to feelings of fatigue and burnout when confronted with job changes. Considering this, the study suggests that hospitals should optimize the night shift system by reducing the number of night shifts, strengthening health management and psychological support, and enhancing the work environment and resource support to alleviate nurses’ change fatigue.

#### Care perception

The results of this study indicated that clinical nurses perceived head nurse care items as generally moderate (6.15 ± 1.07), which aligns with previous survey findings among psychiatric nurses in this group [8]. This similarity suggests potential commonalities in nurses’ perceptions of care across different departments or nursing fields. The change fatigue of clinical nurses was negatively correlated with the perceived level of care (*r* = −0.765, *p* < 0.001) and was significantly affected by the level of perceived head nurse care (*β* = −0.243, *p* < 0.001), indicating that as the level of care provided by head nurses increased, clinical nurses experienced lower levels of fatigue.

Watson, a theorist of human nature and care (18), noted that care is infectious to both participants and observers. By offering care and support, head nurses can foster a more harmonious and supportive working atmosphere, effectively assisting nurses in coping with the pressures of change and reducing their sense of fatigue (19). To mitigate potential harm, management should strengthen communication with frontline nurses. Additionally, timely assistance during critical moments is essential. In addition to establishing a solid, long-term communication bridge, management should actively engage in organizational change, comprehensively control the pace of change, rationally allocate tasks, and have a keen insight into the key stages of fatigue during the change process, allowing for the deployment of preventive measures in advance (20).

Additionally, an extreme case of head nurse support style is micromanagement, which refers to excessive attention to detail and constant oversight. Micromanagement can undermine nurses’ autonomy and well-being, potentially leading to increased work stress and burnout (21). Therefore, while head nurse support is crucial, it is equally important to avoid micromanagement to ensure a supportive and empowering work environment.

Furthermore, management should be vigilant about nurses’ overtime behavior during organizational change and avoid blindly praising such behavior, as this could inadvertently promote the spread of a “super nurse” culture (22).

#### Emotional intelligence

The results of this study revealed that clinical nurses’ emotional intelligence scores were at a medium level (4.65 ± 0.74) (9). This similarity further verifies the stability and universality of emotional intelligence across different studies and samples. Emotional intelligence, defined as an individual’s ability to identify, understand, utilize, and manage their own and others’ emotions, is particularly crucial for nurses, a profession that necessitates frequent interaction with patients and their families (23).

The study found that clinical nurses’ change fatigue was negatively correlated with their emotional intelligence level (*r* = −0.666, *p* < 0.001) and was significantly affected by it (*β* = −0.123, *p* < 0.001). Specifically, for each unit increase in emotional intelligence level, the degree of change fatigue was notably reduced. This further underscores the vital role of emotional intelligence in mitigating change fatigue. Emotional intelligence not only aids nurses in better understanding and regulating their emotions but also facilitates effective communication with colleagues, patients, and their families, thereby reducing the negative emotions and stress associated with change (24).

Furthermore, the potential fatigue-inducing effects of emotional self-regulation should not be overlooked. While emotional intelligence aids in managing stress, excessive regulation of emotions may lead to increased fatigue (4). Additionally, the negative impact of micromanagement on nurses’ autonomy and well-being highlights the importance of balancing support with empowerment in leadership styles (21).

In response to this phenomenon, the study offers the following recommendations: Firstly, organizations should conduct targeted emotional intelligence training to enhance nurses’ emotional recognition, regulation, and social skills. Secondly, they should establish and improve psychological support systems to provide professional psychological counseling and guidance for nurses with lower emotional intelligence (15). Lastly, management should consider nurses’ emotional intelligence levels and adopt more detailed and humanized change management strategies (25).

Based on the findings of this study, we recommend that clinical nurses engage in emotional intelligence training to enhance their ability to manage stress and adapt to change (26). Additionally, nursing managers should avoid micromanagement and instead focus on providing supportive and empowering leadership to improve nurses’ job satisfaction and well-being (10).

## Conclusion

In this study, clinical nurses exhibited moderate levels of change fatigue, with influencing factors encompassing monthly income, professional title, years of experience, number of monthly night shifts, care perception, and emotional intelligence. However, due to constraints related to manpower and time, the impact of varying times on clinical nurses’ change fatigue was not explored. Furthermore, the study only included nurses from a single tertiary hospital, resulting in an underrepresented sample. Additionally, the influencing factors considered may have been inadequate. It is hoped that future studies will expand the sample size and dynamically analyze potential predictors of change fatigue to develop more effective interventions.

## Data Availability

The raw data supporting the conclusions of this article will be made available by the authors, without undue reservation.

## References

[1] Chen AJ Richards MR Shriver R. Fitting in? Physician practice style after forced relocation. Health Serv Res. (2024) 59:e14340. doi: 10.1111/1475-6773.14340, PMID: 38886564 PMC11249821

[2] Beaulieu L Seneviratne C Nowell L. Change fatigue in nursing: an integrative review. J Adv Nurs. (2023) 79:454–70. doi: 10.1111/jan.15546, PMID: 36534455

[3] McMillan K Perron A. Change fatigue in nurses: a qualitative study. J Adv Nurs. (2020) 76:2627–36. doi: 10.1111/jan.14454, PMID: 32542846

[4] Partido BB Owen J. Relationship between emotional intelligence, stress, and burnout among dental hygiene students. J Dent Educ. (2020) 84:864–70. doi: 10.1002/jdd.12172, PMID: 32359093

[5] Griffin SM Howard S. Individual differences in emotion regulation and cardiovascular responding to stress. Emotion. (2022) 22:331–45. doi: 10.1037/emo0001037 34807696

[6] Kwiecień-Jaguś K Wujtewicz M. Multifactorial analysis of fatigue scale among nurses in Poland. Open Med. (2016) 11:593–604. doi: 10.1515/med-2016-0097, PMID: 28352852 PMC5329884

[7] Saif N Amelia GGGG Rubin A Shaheen I Murtaza M. Influence of transformational leadership on innovative work behavior and task performance of individuals: the mediating role of knowledge sharing. Heliyon. (2024) 10:e32280. doi: 10.1016/j.heliyon.2024.e32280, PMID: 38933951 PMC11200340

[8] Kasemy ZA Sharif AF Bahgat NM Abdelsattar S Abdel Latif AA. Emotional intelligence, workplace conflict and job burn-out among critical care physicians: a mediation analysis with a cross-sectional study design in Egypt. BMJ Open. (2023) 13:e074645. doi: 10.1136/bmjopen-2023-074645, PMID: 37898489 PMC10619067

[9] Yu M Wang H Wu Y Zhang Q Du X Huang X. The influence of emotional intelligence on psychiatric Nurses' Care behavior, and the chain mediating role of compassion fatigue and perception of management. J Psychosoc Nurs Ment Health Serv. (2025) 63:35–43. doi: 10.3928/02793695-20241101-02, PMID: 39508679

[10] Wang Y Han T Han G Zheng Y. The relationship among nurse Leaders' humanistic care behavior, Nurses' professional identity, and psychological security. Am J Health Behav. (2023) 47:321–36. doi: 10.5993/AJHB.47.2.12, PMID: 37226340

[11] Ma J Peng W Pan J. Investigation into the correlation between humanistic care ability and emotional intelligence of hospital staff. BMC Health Serv Res. (2022) 22:839. doi: 10.1186/s12913-022-08227-4, PMID: 35773661 PMC9244559

[12] Fernemark H Karlsson N Skagerström J Seing I Karlsson E Brulin E. Psychosocial work environment in Swedish primary healthcare: a cross-sectional survey of physicians' job satisfaction, turnover intention, social support, leadership climate and change fatigue. Hum Resour Health. (2024) 22:70. doi: 10.1186/s12960-024-00955-4, PMID: 39443998 PMC11500482

[13] Sarıgül SS Uğurluoğlu Ö. Examination of the relationships between change fatigue and perceived organizational culture, burnout, turnover intention, and organizational commitment in nurses. Res Theory Nurs Pract. (2023) 37:311–32. doi: 10.1891/RTNP-2023-0018, PMID: 37380219

[14] Brown R Abuatiq A. Resilience as a strategy to survive organizational change. Nurs Manag. (2020) 51:16–21. doi: 10.1097/01.NUMA.0000651180.42231.ef, PMID: 31990857

[15] Chen X Chen M Zheng H Wang C Chen H Wu Q. Effects of psychological intervention on empathy fatigue in nurses: a meta-analysis. Front Public Health. (2022) 10:952932. doi: 10.3389/fpubh.2022.952932, PMID: 36311568 PMC9614432

[16] Damen LJ Van Tuyl LHD Korevaar JC Knottnerus BJ De Jong JD. Citizens' perspectives on relocating care: a scoping review. BMC Health Serv Res. (2024) 24:202. doi: 10.1186/s12913-024-10671-3, PMID: 38355575 PMC10868012

[17] Damen LJ Van Tuyl LHD Knottnerus BJ De Jong JD. General practitioners' perspectives on relocating care: a Dutch interview study. BMC Prim Care. (2024) 25:186. doi: 10.1186/s12875-024-02425-1, PMID: 38796424 PMC11127345

[18] Akbari A Nasiri A. A concept analysis of Watson's nursing caritas process. Nurs Forum. (2022) 57:1465–71. doi: 10.1111/nuf.12771, PMID: 35767362

[19] Compton RM Hubbard Murdoch N Press MM Lowe ME Ottley KM Barlow M. Capacity of nurses working in long-term care: a systematic review qualitative synthesis. J Clin Nurs. (2023) 32:1642–61. doi: 10.1111/jocn.16144, PMID: 34841614

[20] Chachula KM Varley E. Perceptions and experiences of psychological trauma in nursing and psychiatric nursing students: a small scale qualitative case study. PLoS One. (2022) 17:e0277195. doi: 10.1371/journal.pone.0277195, PMID: 36327303 PMC9632886

[21] Lee J Ahn S Henning MA van de Ridder JMM Rajput V. Micromanagement in clinical supervision: a scoping review. BMC Med Educ. (2023) 23:563. doi: 10.1186/s12909-023-04543-3, PMID: 37559079 PMC10410949

[22] Venturato L Horner B Etherton-Beer C. Development and evaluation of an organisational culture change intervention in residential aged care facilities. Australas J Ageing. (2020) 39:56–63. doi: 10.1111/ajag.12667, PMID: 31070297

[23] Urtubia-Herrera V Navarta-Sánchez MV Palmar-Santos AM Pedraz-Marcos A García-Gomez A Luis EO. The relationship between sense of coherence and emotional intelligence as individual health assets for mental health promotion in students and healthcare professionals: a scoping review. Front Public Health. (2024) 12:1304310. doi: 10.3389/fpubh.2024.1304310, PMID: 38450140 PMC10916004

[24] Richards AE Curley KL Zhang N Bendok BR Zimmerman RS Patel NP. Burnout and emotional intelligence in neurosurgical advanced practice providers across the United States: a cross-sectional analysis. World Neurosurg. (2021) 155:e335–44. doi: 10.1016/j.wneu.2021.08.066, PMID: 34425289

[25] Awe AO David-Olawade AC Ayodele-Awe I Feng H Odetayo A Afolalu TD. Predictors and influencing factors of emotional intelligence among nurses in the north East England, United Kingdom. J Educ Health Promot. (2023) 12:236. doi: 10.4103/jehp.jehp_1656_22, PMID: 37727434 PMC10506741

[26] Booher L Yates E Claus S Haight K Burchill CN. Leadership self-perception of clinical nurses at the bedside: a qualitative descriptive study. J Clin Nurs. (2021) 30:1573–83. doi: 10.1111/jocn.15705, PMID: 33555652

